# Supplementary material for: Causal Relationship between Aging and Anorexia Nervosa: A White-Matter-Microstructure-Mediated Mendelian Randomization Analysis
Source: Biomedicines. 2024 Aug 16;12(8):1874. doi: 10.3390/biomedicines12081874 (PMC11351342; doi:10.3390/biomedicines12081874)
Supplement: Supplementary file 1 [file biomedicines-12-01874-s001.zip › Supplementary Figure/Figure S4/Singe SNP plot of MR analysis for TL to ubm-b-1909.pdf]

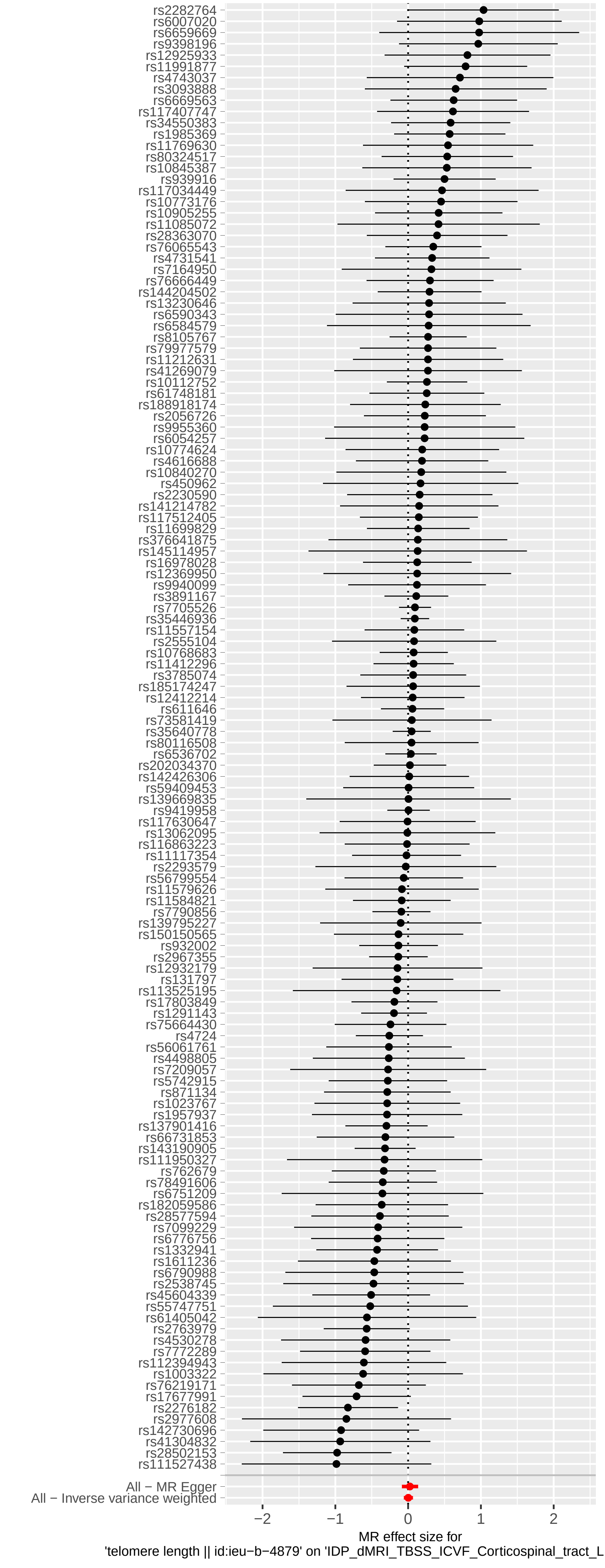
rs2282764
rs6007020
rs6659669
rs9398196
rs12925933
rs11991877
rs4743037
rs3093888
rs6669563
rs117407747
rs34550383
rs1985369
rs11769630
rs80324517
rs10845387
rs939916
rs117034449
rs10773176
rs10905255
rs11085072
rs28363070
rs76065543
rs4731541
rs7164950
rs76666449
rs144204502
rs13230646
rs6590343
rs6584579
rs8105767
rs79977579
rs11212631
rs41269079
rs10112752
rs61748181
rs188918174
rs2056726
rs9955360
rs6054257
rs10774624
rs4616688
rs10840270
rs450962
rs2230590
rs141214782
rs117512405
rs11699829
rs376641875
rs145114957
rs16978028
rs12369950
rs9940099
rs3891167
rs7705526
rs35446936
rs11557154
rs2555104
rs10768683
rs11412296
rs3785074
rs185174247
rs12412214
rs611646
rs73581419
rs35640778
rs80116508
rs6536702
rs202034370
rs142426306
rs59409453
rs139669835
rs9419958
rs117630647
rs13062095
rs116863223
rs11117354
rs2293579
rs56799554
rs11579626
rs11584821
rs7790856
rs139795227
rs150150565
rs932002
rs2967355
rs12932179
rs131797
rs113525195
rs17803849
rs1291143
rs75664430
rs4724
rs56061761
rs4498805
rs7209057
rs5742915
rs871134
rs1023767
rs1957937
rs137901416
rs66731853
rs143190905
rs111950327
rs762679
rs78491606
rs6751209
rs182059586
rs28577594
rs7099229
rs6776756
rs1332941
rs1611236
rs6790988
rs2538745
rs45604339
rs55747751
rs61405042
rs2763979
rs4530278
rs7772289
rs112394943
rs1003322
rs76219171
rs17677991
rs2276182
rs2977608
rs142730696
rs41304832
rs28502153
rs111527438
All − MR Egger
All − Inverse variance weighted
−2
−1
0
1
2
MR effect size for
'telomere length || id:ieu−b−4879' on 'IDP_dMRI_TBSS_ICVF_Corticospinal_tract_L
